# Penetration of Water-Soluble Material through Gas-Cleaning Filters

**DOI:** 10.3390/membranes12080776

**Published:** 2022-08-12

**Authors:** Almuth D. Schwarz, Jörg Meyer, Achim Dittler

**Affiliations:** Institute of Mechanical Process Engineering and Mechanics, Karlsruhe Institute of Technology, Straße am Forum 8, 76131 Karlsruhe, Germany

**Keywords:** surface filtration, salts, water mist, soluble particles, gas cleaning

## Abstract

To predict the behavior of gas-cleaning filters during real-world operation, it is essential to understand their response to ambient conditions. The temporary presence of water droplets in gas-cleaning filtration systems due to fog, spray rain, or condensation, as examples of irregular events, has an impact on the filters’ operating performance, especially when soluble particles are present. In this work, surface filters were loaded with mixtures of water-soluble salt particles and insoluble glass spheres. These were, subsequently, exposed to water mist and dried by a particle-free gas stream. A novel approach to analyze the drainage of solution on filters with soluble filter cakes is presented, which allows the detection of solubles on the clean gas side of the filter. As a result, this work, for the first time, presents a sighting of the penetration of soluble filter cake material through gas-cleaning filters. Furthermore, filter performance, in terms of differential pressure and fractional separation efficiency, was determined and a characteristic differential pressure evolution for hydrophilic filters during exposure to water mist was also identified. The fractional separation efficiency of gas-cleaning filters decreases due to exposure to water mist. The findings are supported by scanning electron microscopy (SEM) images, energy-dispersive X-ray (EDX), and X-ray microtomography (µ-CT analysis) images.

## 1. Introduction

Surface filtration is a well-known process to separate solid particles from a gas stream. At a high relative humidity (RH), the filters’ operating behavior changes concerning the separation efficiency and differential pressure evolution [1,2]. This holds especially true for particle systems containing water-soluble, hygroscopic salts [3,4,5,6,7,8,9,10,11,12]. Salt particles naturally occur in the atmosphere and change their state of matter depending on the ambient RH. When a salt crystal in the ambient air reaches its material-specific deliquescence relative humidity (DRH), the salt absorbs water from its surroundings and dissolves. When the ambient air is subsequently dried and the RH drops below the efflorescence relative humidity, the salt crystallizes. Additional liquid droplets increase the amount of water in the surroundings and, upon exposure, also lead the salt particles to dissolve. Both elevated RH and water droplets in the presence of aerosols with a salt content have been linked to filtration inefficiencies and system failures [13].

The exposure of a surface filter with soluble and/or insoluble filter cake components to water mist can initially lead to the resuspension and dissolving of particles. This mobilization can initiate material transport dominated by flow and capillary forces or even gravity. Additionally, subsequent drying of the filter and crystallization of solubles can lead to a new particle arrangement. Based on these mechanisms, four potential scenarios for the transport of soluble and insoluble material collected on a surface filter during exposure to water mist were identified that could potentially take place:

1. Restructuring of the deposit of soluble and insoluble material on the raw gas surface of the filter medium;
2. Draining of soluble and insoluble material with liquid on the raw gas side of the filter;
3. Penetration of the soluble and insoluble material with the liquid into the filter medium, leading to a structural change of the filter matrix;
4. Penetration of the soluble and insoluble material with the liquid through the full depth of the filter medium, leading to drainage or re-entrainment on the downstream side of the filter.

**Figure 1:**
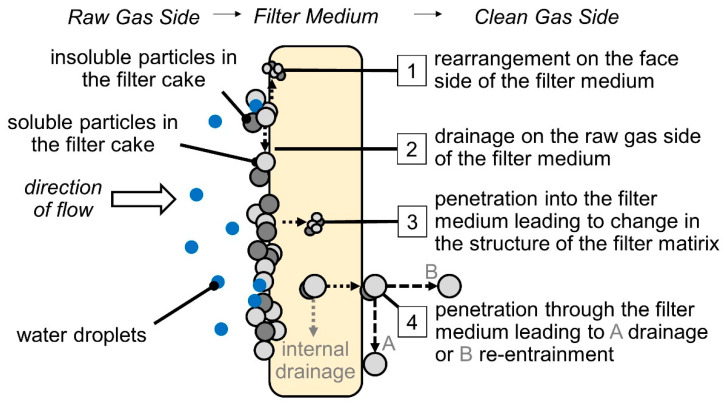
Four scenarios could potentially be initiated by the exposure of a surface filter with soluble and insoluble filter cake components to water mist.

Supplementary internal drainage is typically only to be expected with very thick, open-pored filter media and is, therefore, not considered in detail here. The four mechanisms are depicted in Figure 1.

In previous studies, the authors have analyzed the first scenario with fully soluble filter cakes. The rearrangement of salt particles on the raw gas side of a filter upon exposure to a RH above the salts’ DRH was described as the result of the short-term mechanism of dissolving, which causes a breakup of the filter cake [11,14]. This, in turn, led to an instant decrease in the observed differential pressure. An increase in the amount of supplied water, leading to fully saturated air or even water droplets, enhanced the effects further. This resulted in a more sudden differential pressure decrease even at short exposure to water mist. In this paper, additionally, the rearrangement of particles on the filter surface upon exposure to water mist is investigated for insoluble filter cakes and filter cakes with soluble and insoluble particle mixtures. 

The second stated scenario of originally deposited material draining with liquid on the raw gas side of the medium is unlikely for the material system in this study, but may be relevant to other material systems investigated in the future, and was added for completeness.

In the third scenario, the soluble and insoluble material is considered to penetrate with the liquid in solution or by force of flow, into the void space inside the filter medium. If it remains there during the following drying process, this could cause a structural change of the filter matrix by blocking part of the void space and by changing the size and shape of the collectors. The initial focus in evaluating these scenarios lies in determining the limiting cases, i.e., the ones initiated by short-term and long-term exposure to water mist. The penetration of solution into and crystallizing of salt within the filter medium is likely to be relevant at intermediate exposure times and is, therefore, not covered by this study at this point. 

In the fourth scenario, the penetration of the soluble and insoluble material with the liquid through the full depth of the filter medium is considered. This would result in drainage or re-entrainment on the downstream side of the filter. The analysis of this scenario on filters laden with soluble particles (salt) only, as well as mixtures of soluble (salt) and insoluble (glass) particles is the main subject of this paper. This includes analysis of the liquid drainage, in which the majority of penetrated particle material is expected. The authors have previously stated that the long-term exposure of surface filters with salt filter cakes to water droplets can, in principle, lead to the penetration of initially separated salt particles through the filter medium [15,16,17]. However, this work is the first comprehensive analysis of the penetration of soluble particle material through a filter medium due to exposure to water mist, and is the first consideration of insoluble particles were considered.

## 2. Materials and Methods

### 2.1. Particle System

Potassium Sulfate (K_2_SO_4_) serves as a soluble test dust in the experiments. It has a good solubility in water of 111 g/L at 20 °C, an efflorescence relative humidity of 60% ± 2, and a high DRH of 96% ± 1 [18]. Therefore, the overlapping effect of its deliquescence behavior and dissolution at direct contact with water is kept at a minimum. Additionally, any changes in crystal structure due to deliquescence behavior at varying ambient RH between removing the filters from the test rig and analyzing samples is minimized as well.

Speriglass 5000 CP00 (Potters, PA, USA) serves as an insoluble test dust. 

### 2.2. Filter Medium

A hydrophilic polyester/polyamid spunbonded nonwoven with hydroentangled microfilaments serves as the test filter medium. It has an area weight of 240 g/m^2^ and a thickness of 1 mm. Its median pore diameter is 12 µm ± 1 (determined by capillary flow porometry) and its air permeability at 200 Pa is 100 L/min·dm^2^. Round filter coupons with an active filtration area of 100 cm^2^ are used in the experiments. The coupons were pre-selected depending on the initial differential pressure Δp_0_. All employed filters have a Δp_0_ at 3.5 cm/s of 0.48 mbar ± 0.12.

### 2.3. Experimental Set-Up

**Figure 2:**
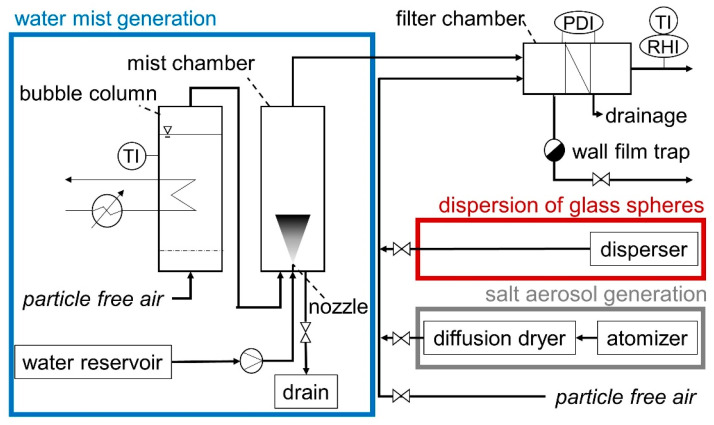
Experimental setup including particle generation, water mist generation, and filter chamber.

The experimental setup is depicted in Figure 2. The test rig is in a temperature-controlled room at 20 °C and comprises three sections:

- The particle generation for the filter cake formation;
- The water aerosol generation;
- The filter chamber.

**Figure 3:**
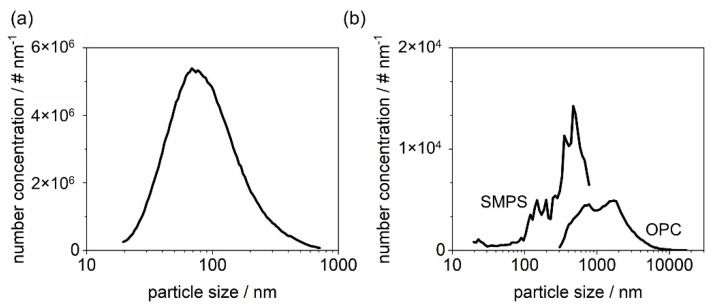
Particle size distribution of K_2_SO_4_ measured with an SMPS (**a**) and glass spheres measured with SMPS and OPC (**b**).

The filter cake is formed with K_2_SO_4_ particles or glass spheres or a mixture of both materials. The salt aerosol is generated by atomization (AGK 2000, Palas GmbH, Karlsruhe, Germany) of a 10 g/L K_2_SO_4_ solution and subsequent drying of the droplets in motion by a diffusion dryer (DDU 570/H, Topas GmbH, Dresden, Germany). For all particle loads, the operating parameters of the atomizer remain the same with a flow rate of 5 L/min to maintain a constant particle size distribution (Figure 3a). The particle size distribution is measured with a scanning mobility particle sizer, SMPS 3938 (TSI GmbH, Aachen, Germany). The glass spheres are dispersed with an RBG 1000 (Palas GmbH, Karlsruhe, Germany). The particle size distribution of the glass spheres entering the filter chamber was measured with an SMPS 3938 (TSI GmbH, Aachen, Germany) up to a size of 800 nm and an optical particle counter (OPC) Promo 3000 H (Palas GmbH, Karlsruhe, Germany) up to 18 µm (Figure 3b).

To generate a stable, finely dispersed water phase, an initially dry, particle-free air stream passes through a bubble column and is, thus, saturated. To control the temperature of the bubble column, it is equipped with a thermostat (CC-K12, Peter Huber Kältemaschinenbau AG, Offenburg, Germany) and a thermocouple. The saturated air stream passes through a water mist chamber where fine droplets are produced by atomizing demineralized water with a full cone nozzle (Micro-line Nebeldüse 0.1 mm, Micro Rain Systems e.K., Altrenburg, Germany) operated at 70 bar. Since the air stream is saturated, the particle distribution of the water droplets can be assumed to remain constant with a number-based median value of x_50.0_ ≈ 3 µm as provided by the nozzle supplier. Water droplets, deposited on the walls of the water mist chamber drain into a water tank. The remaining water droplets are carried to the filter chamber by the saturated air stream with a volumetric flow rate of liquid water of 0.050 mL/min. The flow rate was estimated by measuring the change in mass of the hydrophilic medium, which was exposed to the water mist over a defined time period.

The filter chamber, designed specifically for this application, is equipped with a wall film trap, a groove with a protruding ridge around the active filtration area leading the water film on the walls to a drain, immediately upstream of the filter media. Additionally, a drain is placed immediately downstream of the filter. Temperature and humidity sensors (EE08, E + E Elektronik, Engerwitzdorf, Germany) are installed in the exhaust air stream downstream of the filter chamber, and the differential pressure across the filter is recorded (Wet/Wet Diff. Pres. Trans., Omega Engineering, Deckenpfronn, Germany).

### 2.4. Procedure

For a full discharge of the filter coupons prior to usage, a method based on ISO 16890-4:2020 was applied. Each filter coupon is saturated by diffusion over a period of 2 h in a sealed Isopropanol saturated container. Subsequently, the filter coupons dry in a conditioned room at 40% RH and a temperature of 20 °C for at least 24 h. 

The experiments are carried out at 20 °C. In each step of the experiment, a constant sensible face velocity for surface filters of 3.5 cm/s is maintained. 

Each experiment contains three steps: particle loading, exposure to water mist, and drying, all followed by post-experimental analysis.

#### 2.4.1. Particle Loading

**Figure 4:**
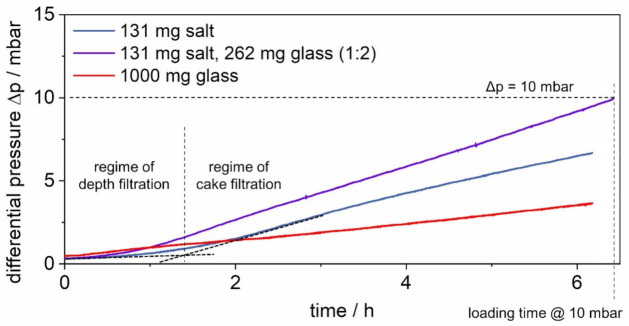
Differential pressure evolution during characteristic particle loading experiments of the salt-laden, the salt–glass-laden (ratio 1:2), and the glass-laden filter.

In the first step of the experiments, all particles enter the filter chamber in a dry gas stream at an RH below 15%. The filters are loaded with either soluble salt, salt and insoluble glass, or glass. Figure 4 shows the differential pressure evolution during three characteristic particle loading experiments for different aerosol compositions (results of single experimental runs). 

The lowest stable salt–glass ratio with the disperser at hand is 1:2. This ratio is used to load the filter up to a common final differential pressure for the regeneration of surface filters of 10 mbar. For multiple repetitions, Δp = 10 mbar was reached after 6.2 h on average, resulting in a loading of 131 mg salt and 262 mg glass.

To allow for a direct comparison of the salt amounts on the filters and in the drainage later on, the salt-laden filter is loaded over the same average loading time of 6.2 h. This corresponds to the same total mass loading of 131 mg of salt. During salt loading, the generated aerosol is diluted with particle-free air to reach the face velocity of 3.5 cm/s. 

The loading time of 6.2 h was also used for the glass-laden filter. However, a higher mass load of 1000 mg compared to the 262 mg on the salt–glass-laden filter was chosen. This allows for a more distinct increase in the differential pressure.

The mass flow rate of the glass spheres was determined gravimetrically over 2 h for each disperser setting individually right before the experiment. 

As indicated with auxiliary tangents in Figure 4, the loading time of 6.2 h corresponds to particle loading in the regime of surface filtration for all filters.

When the particle loading is stopped, the filter is continued to be exposed to a constant flow of dry particle free air at RH < 10%, to maintain a stable filter cake and no exposure to elevated RH until the start of the exposure to water mist. This operating step is not shown in the differential pressure plots.

#### 2.4.2. Exposure to Water Mist

During the second step of the procedure, the particle-loaded filter is exposed to water mist. As liquid penetrates the filter medium and reaches the clean gas side of the filter where it drains. While the filter is exposed to water mist, the drainage is captured in individual aluminum dishes over a period of 65 min. To obtain information about possibly penetrated soluble or insoluble material, which was initially separated in the filter cake, the drainage is analyzed according to the following procedure:

Before use, the dishes are washed with water and isopropanol, dried at 100 °C, and conditioned in a room at 40% RH and a temperature of 20 °C. This removes any contaminants to ensure stable, reproducible conditions for precise weight measurements. Each dish is weighed before and after collecting the drainage. The samples with drainage are then heated up to 100 °C to evaporate the water. After conditioning at 40% RH and 20 °C, the mass of the remaining penetrated soluble and insoluble material in each sample is obtained. Rinsing the dishes produces a solution, which is filtered through a PC-membrane filter with a pore size of 0.2 µm. The difference in weight of the membrane before and after the experiment is the weight of insoluble material that has passed through the filter. The aluminum dishes are, finally, again washed with water and isopropanol and conditioned at 40% RH and 20 °C to verify that their weight remained constant throughout the whole procedure.

#### 2.4.3. Drying

The third and final step is initiated after visible drainage over 65 min. All filters are dried with a particle-free air stream with RH < 10% before any further analysis. 

#### 2.4.4. Post-Experimental Analysis and Reference Filters

Following the experiment, all filter coupons are conditioned at 40% RH and a temperature of 20 °C for a period of 24 h, before they are weighed. Samples are then examined using scanning electron microscopy (SEM), energy-dispersive X-ray (EDX), and X-ray microtomography (µ-CT analysis). For SEM and EDX analysis filters were cut to 1 cm^2^ samples and sputter-coated by a 4.5 nm thick palladium layer. µ-CT scans presented in this publication were generated with a ZEISS Xradia 520 Versa. A detailed description of its functionality and use can, for example, be found in the work by Straube et al. [19]. A sample of 0.5 cm × 2 cm was cut out of the filter. Since salt could be mobilized and salt crusts could be broken by the act of cutting the filter, the sample size was chosen to be larger than the scan area. Due to the stability of the filter medium itself, no support structure had to be employed. After a warm-up scan to avoid thermal expansion, the main scan of the full depth of the filter reached a voxel size of 1.02 µm and a detail-scan of the center of the filter reached a voxel size of 0.67 µm. Since objects in the size of approximately 10 times the voxel size can be segmented appropriately, the minimal resolution is roughly 6.7 µm. For the segmentation of the reconstructed stack of 2D images from the µ-CT, the software Dragonfly (V. 2021.2 for Windows) (Object Research Systems (ORS) Inc., Montreal, CA, USA) is used. After segmentation with the deep learning tool in Dragonfly, small traces of material (close to the border of minimal resolution) were individually looked at to differentiate between traces of salt or artifacts.

No filters were removed from the filter chamber during the experiments, thus protecting them from uncontrolled moisture variations. To estimate the initial load of the filters after particle loading, an additional set of filters is prepared and loaded according to the first procedure step. These reference filters are then removed from the test rig after particle loading. After conditioning at 40% RH and 20 °C over 24 h, the mass of the filter cake is determined gravimetrically, and reference SEM images are produced of the filter cake, characterizing the state of the filter before exposure to water mist.

An additional set of filters is prepared and undergoes the same procedure with added measurements of the fractional separation efficiency. The fractional separation efficiency is defined in terms of concentration C and particle size x:

fractional separation efficiency = (C(x)_raw gas_ − C(x)_clean gas_)/C(x)_raw gas_ (1)

**Figure 5:**
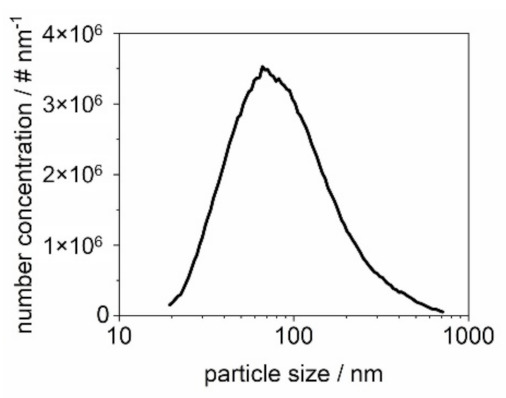
Particle size distribution of the K_2_SO_4_ of the raw gas side used for fractional separation efficiency measurements.

In this work, the fractional separation efficiency of the filters is measured using an SMPS 3938 (TSI GmbH, Aachen, Germany). To allow for a sufficiently high number concentration of ≥100 particles/cm^3^ in each size class, a K_2_SO_4_-aerosol was generated by atomizing a 20 g/L solution at a volumetric flow rate of 4 L/min diluted with particle-free air to reach the face velocity of 3.5 cm/s. The SMPS is installed on the clean gas side of the filter chamber. Figure 5 shows the particle size distribution of the raw gas side.

For the raw gas measurement of the particle size distribution, the test aerosol passing the filter chamber is measured without an installed filter. The clean gas side is measured three times during the experiment, before filter loading, after filter loading, and after exposure to water mist with subsequent drying of the filter. During the exposure to the test aerosol, the filters are loaded with 2.0 g/m^2^ of K_2_SO_4_. Using an additional set of filters for the fractional separation efficiency measurements precludes interference of this additional salt with other results (SEM images and differential pressure).

## 3. Results and Discussion

Filters were loaded with soluble and insoluble particles and exposed to water mist to show and analyze the penetration of the initially separated material. The investigations include differential pressure recordings during the experiments, the gravimetrical analysis of the liquid drainage leaving the filter, fractional separation efficiencies, SEM, EDX, and µ-CT analysis. In this section, representative results of single experiments are presented.

### 3.1. Penetration of Soluble Filter Cake Material

**Figure 6:**
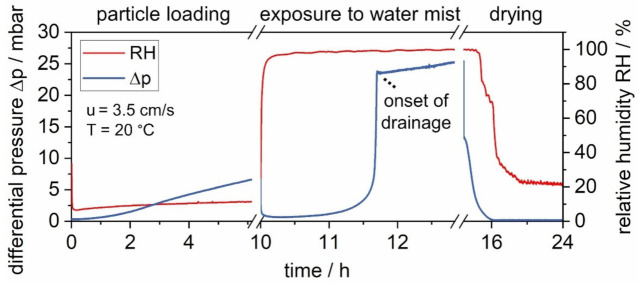
Evolution of differential pressure and RH evolution during particle loading with soluble salt, exposure to water mist, and drying.

The characteristic differential pressure and RH evolution during each step of the experiment are shown in Figure 6 on the example of a salt-laden filter. 

**Figure 7:**
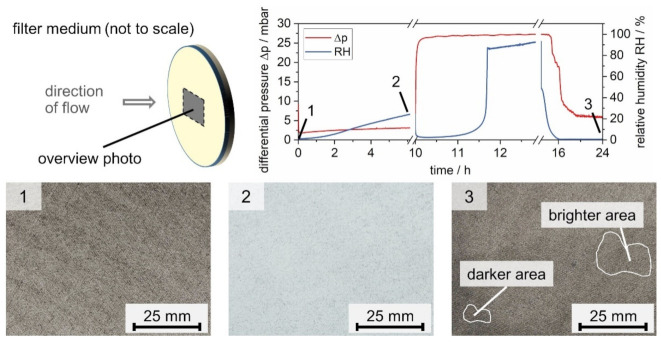
Photos of the upstream side of an unloaded filter (**1**) and salt-laden filters before exposure to water mist (**2**) and after exposure to water mist and drying (**3**).

During the filter loading with solids, the differential pressure increases as particles are separated. As shown in Figure 6, the linear increase towards the end of particle loading indicates operation within the regime of surface filtration. Exposure to water mist leads to a prompt decrease in the differential pressure followed by a slow increase up to approximately 5 mbar. A steep increase follows. At approximately 22 mbar, the differential pressure reaches a local maximum marking the start of drainage. Following the onset of drainage, the differential pressure levels out with a slightly increasing trend. Upon drying, the differential pressure decreases to a value close to the initial Δp_0_. Exposure to water mist is the central step in the experiments. The initial decrease in differential pressure is caused by the restructuring of the (particle) material and the breaking up of the filter cake. This process has been previously described in detail for short-term exposures to water mist by the authors [11,14]. Figure 7 shows three photos: an unloaded filter medium, a salt-laden filter medium before and after water exposure, and subsequent drying. The photos correspond to three points in the experiment, indicated on the differential pressure plot in Figure 7.

**Figure 8:**
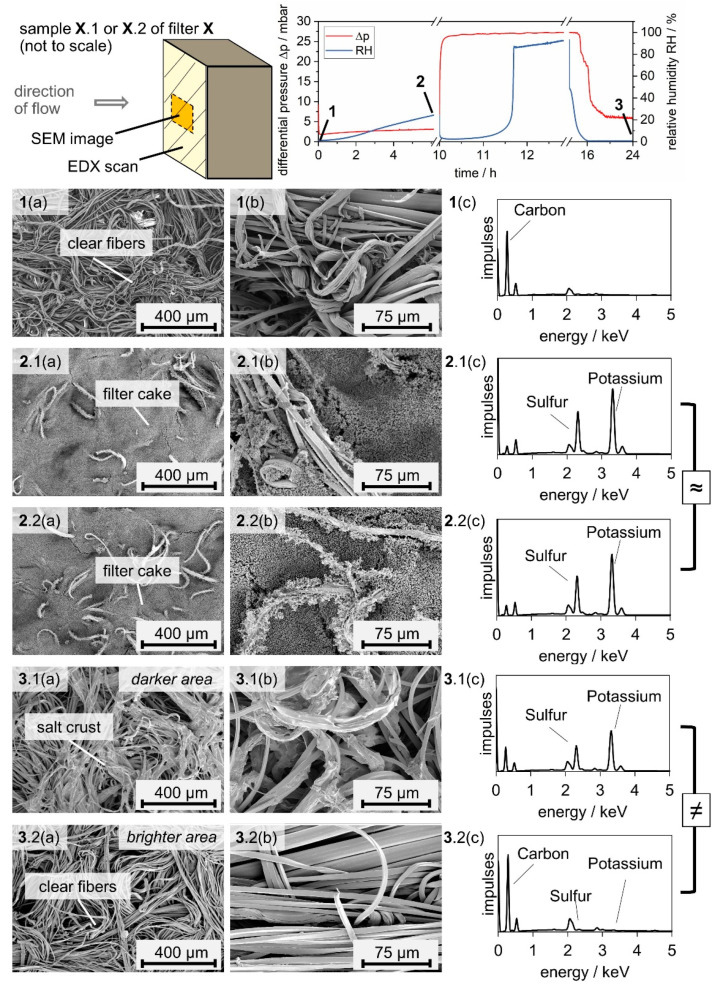
SEM images in small scale, magnification factor ×100 (**a**) and large scale, magnification factor ×500 (**b**) and relevant full-sample EDX area scans (**c**) of one sample before particle loading (**1**) and of two samples after particle loading (**2.1**,**2.2**) and after water exposure and drying (**3.1**,**3.2**).

Figure 7 reveals that the unloaded and salt-laden medium before water exposure look uniform, while the filter medium after exposure to water mist shows brighter and darker areas. SEM images allow correlating the appearance uniformity to the material distribution on the filters. Figure 8 shows the differential pressure diagram of the salt-laden filter with three marked positions: before particle loading (1), after particle loading (2), and after water exposure and drying (3). SEM images in two scales and full-sample EDX scans are presented of the upstream side of samples from filters at the relevant positions. 

**Figure 9:**
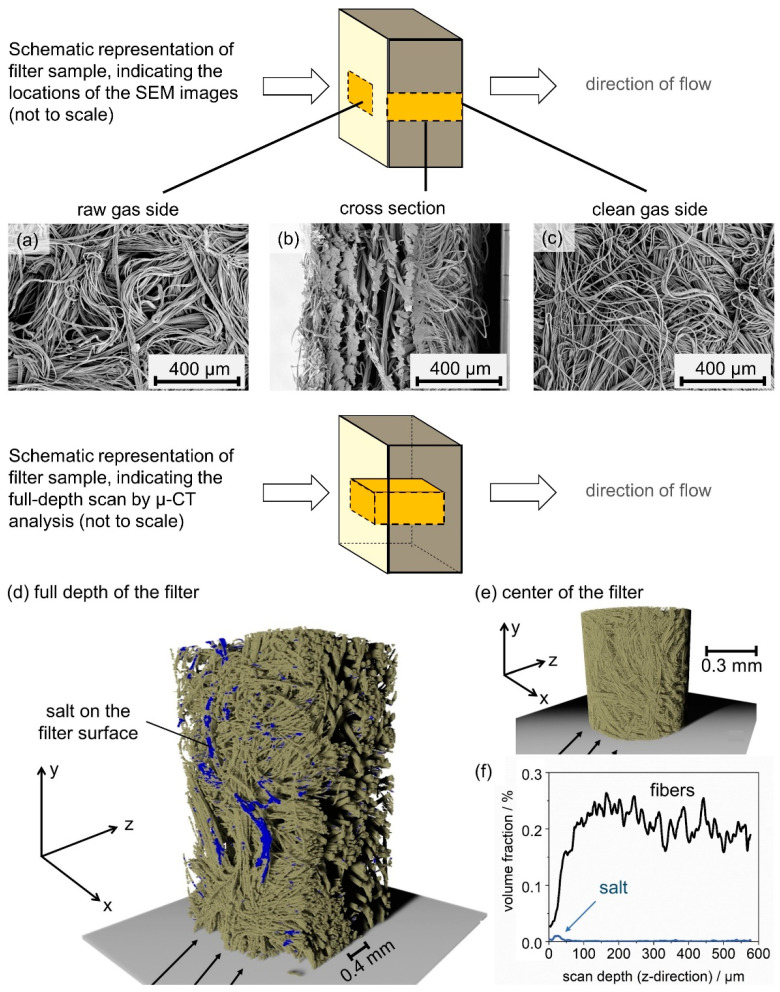
Analysis of a salt-laden filter after exposure to water mist and drying: SEM images of the raw gas side (**a**), cross section (**b**) and clean gas side (**c**), 3D view of a µ-CT scan of the full depth of the filter (**d**), the center of the filter (**e**) and the volume fraction of fibers and salt over the depth of the depicted full-depth µ-CT scan (**f**).

The EDX scan of the unloaded filter medium (Figure 8(1c)) reveals a high carbon content, as expected. After particle loading, the filter is covered with a filter cake (Figure 8(2.1a,2.2a)). The larger scale reveals that the filter cakes are formed of near-spherical particles forming dendrites (Figure 8(2.1b,2.2b)). Both samples (Figure 8(2.1,2.2)) show individual fibers reaching out of the filter cake, which are on both samples partly covered with dendrite structures. According EDX scans support the impression of an evenly formed filter cake on the medium surface (Figure 8(2.1c,2.2c)). After exposure to water mist, the filter cake does not stay intact, which causes a prompt decrease in the differential pressure. The salt is spread unevenly on the filter medium (comp. Figure 8(3.1–3.2)). The darker and brighter area spotted on a photo of the filter (Figure 7) correspond to areas with significant remaining salt (Figure 8(3.1)) and no visible remaining salt (Figure 8(3.2)), respectively. The salt remaining on the surface of the filter crystallized around fiber bundles during the final drying step (Figure 8(3.1b)). This crystallization behavior is known for salt on hydrophilic fibers [20]. The EDX scan shows high peaks for sulfur and potassium, an indicator of high K_2_SO_4_ content. The brighter area has no visible salt and EDX scans confirm that the bright areas of the filter resemble an unloaded filter (comp. Figure 8(1c–3.2c)). Following exposure to water mist and dying, the cross-section of the salt-laden filter was analyzed further using SEM and µ-CT (Figure 9).

No salt was detected in the cross-section or on the clean gas side of the salt-laden filter using SEM (Figure 9b,c). The segmented µ-CT scan of the full depth of the filter, Figure 9d shows salt on the raw gas side of the filter, only. Remaining artefacts on the edges of the scan have been individually analyzed and identified to be not salt (see Section 2.4.4). A scan of the center of the sample with a minimum resolution of approximately 6.7 µm shows no salt (Figure 9e). Additionally, the volume fraction of fibers and salt over the depth of the filter (Figure 9f), calculated from the number of pixels for the whole image stack of the full depth scan (Figure 9d), shows that the salt remaining in the filter after exposure to water mist and drying remains on the surface of the filter medium, more specifically within a surface layer with a thickness of 100 µm. With a minimum resolution of approximately 6.7 µm in the µ-CT analysis and a maximum sample size of up to 1 cm^2^ in the SEM, both methods, SEM and µ-CT analysis, are limited in the resolution of fine salt crusts and sample size. Therefore, the findings presented in Figure 9 do not allow the full exclusion of salt crusts on fibers within the filter medium. However, it is a strong indicator that the majority of salt remaining in the filter after the experiment is on the raw gas side of the filter. 

**Figure 10:**
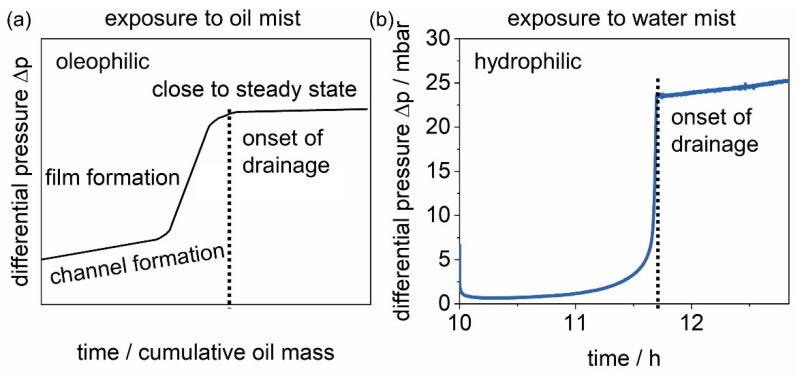
Comparison of (**a**) the schematic differential pressure for oleophilic filter media described by the jump-and-channel model and (**b**) the observed behavior during exposure to water mist of a salt-laden filter (compare Figure 3).

Following the sudden decrease in the differential pressure caused by breaking of the filter cake, the differential pressure evolution closely resembles observations from the related research field of oil droplet separation. Although the material system in this research area is very different from the one investigated, there are strong parallels, namely the separation of liquid droplets from gas streams. In Figure 10, the schematic differential pressure of an oleophilic filter during exposure to oil mist according to the jump-and-channel model (a) is compared to the differential pressure of the salt-laden hydrophilic filter during exposure to water mist (b).

**Figure 11:**
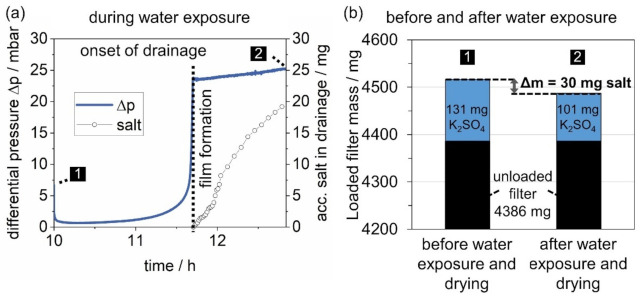
(**a**) Differential pressure of the salt-laden filter during exposure to water mist with the ac-cumulated mass of salt detected in the drainage in relation to (**b**) the gravimetrical analysis of the filter.

The jump-and-channel model [21,22], developed for oil mist filters, states that liquid droplets are collected mainly on the upfront fibers of oleophilic filters and coalesce into larger chunks of liquid, which then make their way into the filter. The differential pressure increases as the liquid forms channels through the medium. As it reaches the clean gas side it forms a liquid film at the interface, which leads to a steep increase in the differential pressure, a “jump”. This increase corresponds to the capillary exit pressure of the oil mist filter. Following film formation, the liquid drains, and the differential pressure reaches a close-to-steady-state (still subject to research [23]). A comparison to the differential pressure of a hydrophilic surface filter during exposure to water mist indicates that, similarly, a liquid film forms downstream of the filter before the process reaches a close-to-steady-state. As the jump-and-channel model suggests, drainage is observed following the film formation. The gravimetrical analysis of the drainage (a) and the filter (b) is related to the differential pressure of the salt-laden filter in Figure 11.

After film formation and onset of drainage, as the exposure to water droplets continues, the cumulative amount of salt leaving the filter in the drainage increases. In total, 20 mg of salt was recovered from the drainage. Overall, the weight of the loaded filter was reduced by 30 mg, due to penetrated salt. It was considered that the filter itself could lose weight during exposure to water mist, e.g., due to loose fibers. However, unloaded filters undergoing the same procedure did not lose weight throughout the experiment. It is possible that the discrepancy between the amount of salt, which left the filter, and the amount of salt, which was recovered from the drainage, is a result of dead zones between the downstream side of the filter and the drainage outlet and re-entrainment. 

This section showed and discussed the fourth predicted penetration scenario (see Section 1): the penetration of the soluble material with the liquid through the full depth of the filter medium, leading to drainage on the downstream side of the filter. The liquid in the wall film trap (located on the raw gas side immediately in front of the filter medium) was analyzed according to the procedure developed for drainage samples. It revealed that the second scenario, i.e., draining of soluble and insoluble material with liquid on the raw gas side of the filter, did not occur during the experiments with the material system at hand.

### 3.2. Penetration of Soluble Material through the Filter Depending on the Amount of Insoluble Material in the Filter Cake

**Figure 12:**
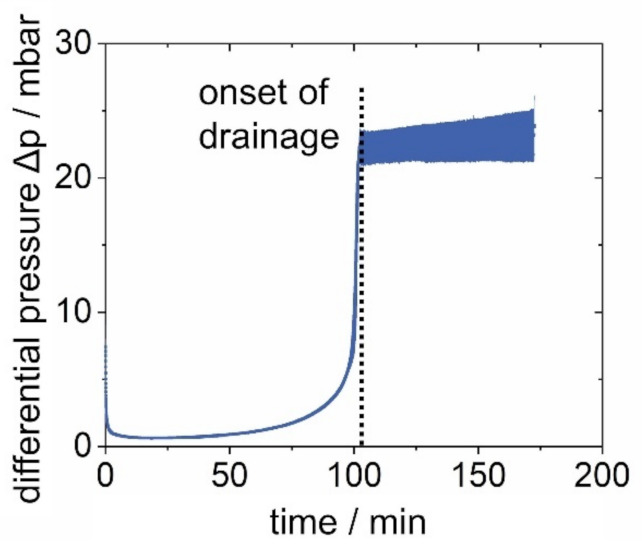
Area of the differential pressure of the salt-laden filter with standard deviation during exposure to water mist.

Filters with different loading rations in soluble and insoluble particles were exposed to water mist. To allow an estimation of the data presented for individual filters, the differential pressure of the salt-laden filter discussed in Section 3.1 is plotted in Figure 12 with standard deviation (filled in area).

**Figure 13:**
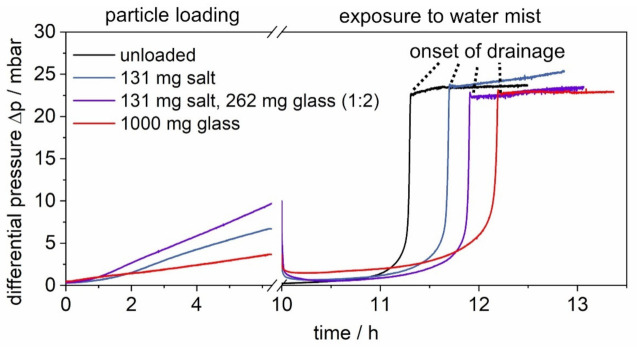
Differential pressure plots during the exposure with water mist of an unloaded filter, a salt-laden filter, a filter loaded with a mixture of salt and glass particles, and a glass-laden filter.

The sharp increase in differential pressure occurs almost simultaneously for all tested filters. The height of the jump, however, shows deviations of up to more than 3 mbar with an increasing tendency after the start of the drainage. This difference in drainage and the subsequent instability is known from oil mist filtration and remains part of current research [23]. Due to the excellent repeatability shown in Figure 12, individual tests will continue to be presented in the following for the sake of clarity. The differential pressure plots of four filters during exposure to water mist are shown in Figure 13:

- Unloaded;
- Loaded with salt, only (131 mg);
- Loaded with salt (131 mg) and glass (262 mg) in a 1:2 ratio;
- Loaded with glass, only (1000 mg).

The differential pressure during particle loading varies, depending on the particle size distribution. The low differential pressure of the glass-laden filter, despite the highest total particle loading in count and weight, results from a lower flow resistance due to much larger particles in the filter cake. The large particle load of the glass-laden filter was chosen to enhance the visibility of effects during exposure to water mist. Upon exposure, the differential pressure of all filters decreases to below 2.5 mbar, caused by a breaking of the filter cake (discussed in Section 3.1). However, the differential pressure of the glass-laden filter remains well above that of the other filters, as well as its initial differential pressure. Unlike the filters loaded with fewer particles, including salt, the glass spheres on the glass-laden filter remain to increase the differential pressure, which is attributed to more material on the filter surface. Additionally, small holes could have formed in the filter cake due to a change in adhesion forces caused by the exposure to water mist, which would reduce the active filtration area, increase the flow velocity, and also result in a higher differential pressure. Unfortunately, due to the large amount and loose structure of the glass spheres on the filter, which hinders appropriate coating and electron flow during SEM sample preparation, it is not possible to provide SEM images for the glass-laden filter to support this hypothesis. Furthermore, the differential pressure plots show that the time of film formation depends on the particle load of the filter. During exposure to water mist, the differential pressure of the unloaded filter shows the earliest “jump” due to film formation. The results show that an initial dust cake on the surface of the filter slows the penetration of liquid down. The delay in the onset of the drainage increases with an increasing particle load. Since slight differences in the jumps are known from the oil mist filtration, and the final differential pressures of the “jumps” all lie within the previously discussed error of 2 mbar (Figure 12), their specific heights and final gradients are not discussed further.

**Figure 14:**
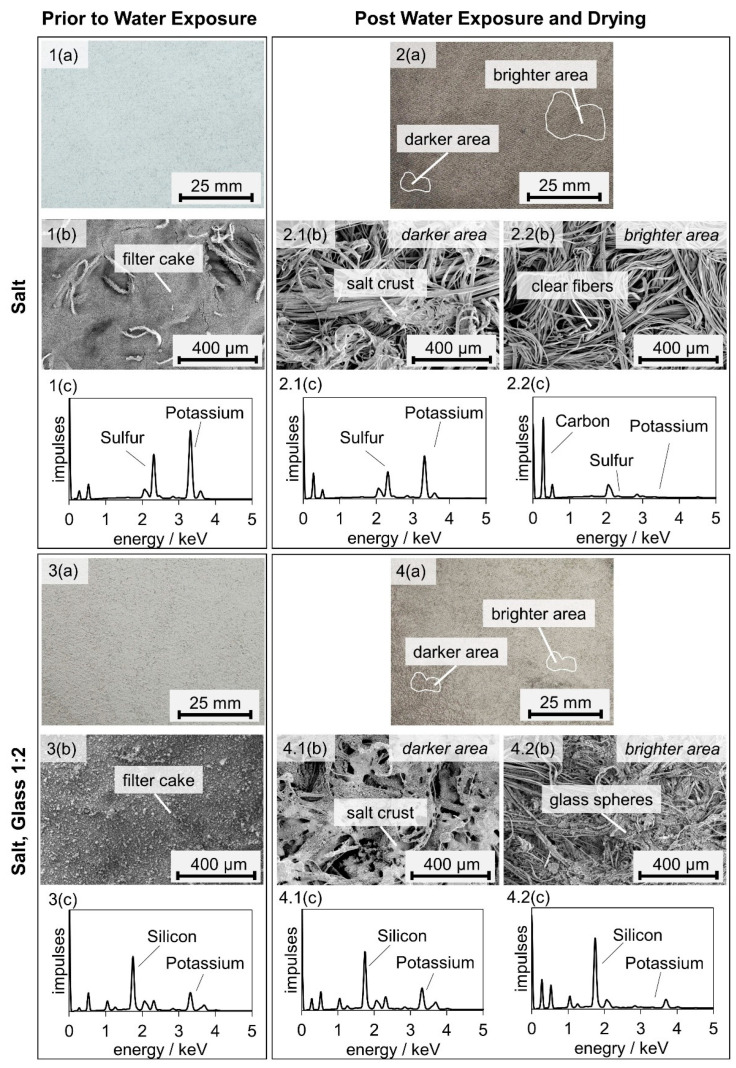
Photos (**a**), SEM images (**b**) and full-sample EDX area scans (**c**) of the raw gas surface of the salt-laden filter before (**1**) and after (**2**) water exposure and drying, and the filter loaded with a mixture of salt and glass particles before (**3**) and after (**4**) water exposure and drying.

To gain a better understanding of the influence of the insoluble particles on the overall material distribution on the filter surface, the filters are analyzed using SEM. In Figure 14, photos and SEM scans of the filter loaded with a mixture of salt and glass particles are compared to the salt-laden filter.

Before exposure to water mist, both filters are evenly covered with a filter cake (Figure 14(1a,3a)). In the case of the salt-laden filter, individual fibers are sticking out of the filter cake, indicating that this filter cake has a smaller thickness (Figure 14(1b)). After exposure to water mist, the salt formed crusts around individual fiber bundles of the salt-laden filter (comp. Figure 8). On the filter with salt and glass, both glass spheres and salt remained on the face side after exposure to water mist. Similar to the salt-laden filter, the salt and glass laden filter showed brighter and darker areas after water exposure and drying (Figure 14(2a,4a)). The darker area shows glass spheres and salt on the SEM image and EDX scan (Figure 14(4.1b,4.1c)), while the brighter area contains predominantly glass spheres/Silicon (Figure 14(4.2b,4.2c)). The material formed patches on the filter surface, which are independent of their position within the test rig, but depend on the individual filter medium only. Exposure to water mist leads to an uneven distribution of glass spheres and salt. IN a comparison of Figure 14, 2.1 and 2.2 with 4.1 and 4.2 shows that more fibers remain covered on the salt- and glass-laden filter. It appears that more particles remain on the salt- and glass-laden filter, and that the salt does not only form crusts around the fiber bundles, but also around the spheres as well, covering more surface area. 

**Figure 15:**
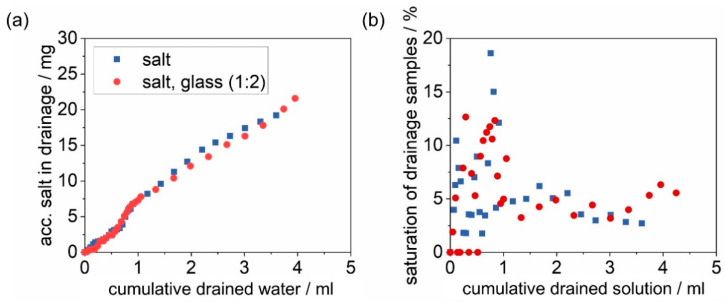
Results of the gravimetrical analysis of the drainage of a salt-laden (initially loaded with 131 mg salt) and a salt–glass-laden filter (initially loaded with 131 mg salt and 262 mg glass), where time = 0 marks the start of the drainage: (**a**) the accumulated salt per cumulative drained water and (**b**) the saturation of drainage samples.

The total amounts of salt and glass recovered from the drainage of each filter are shown in Table 1. No glass was recovered from the drainage of any of the filters. For both the unloaded filter and the glass-laden filter, no particle residues were detected in the drainage. Figure 15 provides the analysis of the drainage of the filters loaded with salt and salt and glass in a ratio 1:2. 

The dissolved salt penetrated the whole depth of the filter medium and drained on the clean gas side. The penetrating amount of salt increases almost in a linear fashion over the 65 min of drainage collection. The insoluble material on the salt–glass-laden filter does not seem to impact the amount of salt penetrating the filter. The saturation of the drainage shows a high variance. In the first 5 mL of drained water leaving the salt–glass-laden filter, no salt was detected. This is another indicator that the salt spread unevenly within the solution on the filter and the water finds preferred pathways, not necessarily coming in contact with any salt. Overall, the saturation of the drainage is constantly below 20%. 

**Figure 16:**
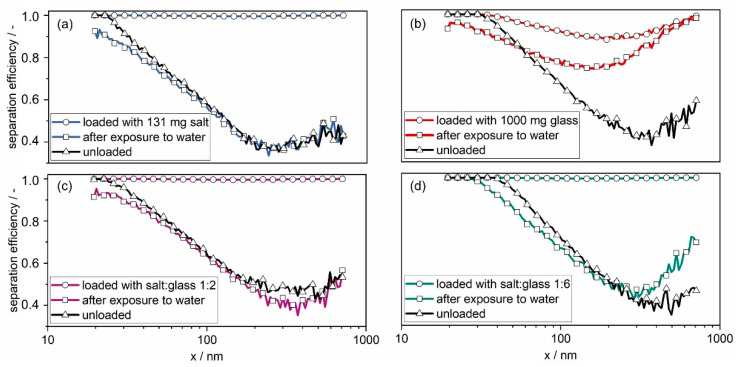
Fractional separation efficiencies before particle loading (unloaded), after particle loading (loaded) and after exposure to water mist of a (**a**) salt-laden, (**b**) glass-laden, (**c**) salt–glass-laden 1:2 and (**d**) salt–glass-laden 1:6 filter. The filters containing salt are all loaded with the same salt mass of 131 mg.

The penetration of salt and the rearrangement of particles on the raw gas side of the filter does not only influence the filter operating behavior in terms of differential pressure, but also the separation efficiency of the filters. Figure 16 shows the fractional separation efficiency of four filters before particle loading, after particle loading, and after exposure to water mist:

- Loaded with salt, only (131 mg);
- Loaded with glass, only (1000 mg);
- Loaded with salt (131 mg) and glass (262 mg) in a 1:2 ratio;
- Loaded with salt (131 mg) and glass (786 mg) in a 1:6 ratio.

The filter loaded with salt (131 mg) and glass (786 mg) in a 1:6 ratio was added to highlight the effect of large amounts of insoluble, non-penetrating particles in the filter cake.

The unloaded filter’s most-penetrating particle size (MPPS) is between 200 and 400 nm, where the separation efficiency is around 40%. Apart from the glass-laden filter, the loaded filters have a separation efficiency above 98% for all particle sizes. The loaded glass-laden filter (Figure 16b) has a lower separation efficiency, remaining just above 87%. The exposure to water mist leads to a decrease in the separation efficiency, caused by the collapse of the filter cake. Figure 16a and c show that the salt-laden and the salt–glass-laden 1:2 filter reach values close to those of the unloaded filter. The MPPS of the glass-laden filter decreases, while the separation efficiency remains above 70% (Figure 16b). The MPPS of the salt–glass-laden 1:6 filter also decreases and its separation efficiency decreases to almost 40% (Figure 16d). The decrease in the MPPS with larger glass content is likely a result of the remaining glass spheres on the raw gas side of the filter, as seen on the SEM images (Figure 14). The active filtration area is thereby reduced; therefore, the face velocity increases. The separation by diffusion decreases at a higher velocity, while the inertial separation and separation by interception increases, thus moving the MPPS to lower particle sizes. A comparable effect is known from filters after patchy cleaning [24], where part of the filter cake remains on the filter, creating patches with lower flow resistance. The reduction in the active filtration area causes an increase in the face velocity. 

## 4. Conclusions

Based on the example of hydrophilic surface filters loaded with either K_2_SO_4_ or a mixture of soluble K_2_SO_4_ and insoluble glass spheres, the penetration of initially collected soluble material through the full depth of gas-cleaning filters was shown for the first time. The penetration is initiated by exposure to water mist, causing the salt to dissolve and penetrate with the water as a solution. The analysis of the drainage revealed that soluble material penetrates the filter medium in a solution, while for the material system at the operating parameters at hand insoluble particles do not penetrate the filter. Analysis of the differential pressure revealed that a short-term exposure to water mist leads to the breaking of the filter cake, causing a sudden decrease in the differential pressure; the corresponding structural changes of the deposit have been shown using SEM. This supports previous findings of the authors [11,14]. For the following long-term exposure to water mist and penetration of the solution, parallels to oleophilic coalescence filters, as described by the jump-and-channel model, have been deduced. The liquid is transported through the filter medium by forces induced by the gas flow and ultimately forms a film of draining liquid on the clean gas side of the hydrophilic filter. This film formation results in a steep increase in the differential pressure. Longer exposure to water mist leads to more salt reaching the clean gas side (and leaving the filter via drainage or re-entrainment). After drying, SEM analysis indicates that the remaining salt forms crusts around the hydrophilic fiber bundles on the raw gas side of the filter, and, additionally, on any initially separated glass spheres in the case of collected particle mixtures. Further combination of SEM and EDX analysis also shows that the initial homogeneous layer of deposited material on the surface of the filter is unevenly distributed after exposure to water mist (no regularity or pattern could be established). SEM and state of the art µ-CT scans indicate that the salt and glass spheres remaining in the filter after exposure to water mist are located on the raw gas surface of the filter, where they were initially separated. A comparison of differential pressure of an unloaded, a salt-laden, a salt-and-glass-laden, and a glass-laden filter shows that with increased particle load, the penetration of the liquid through the filter is slowed, while the film formation kinetics remain unchanged. Fractional separation efficiencies were measured of the filters prior to particle loading, after loading, and after exposure to water mist and drying. The exposure of a loaded filter to water mist leads to a reduction in the fractional separation efficiency. Filters loaded with a high salt ratio and little glass showed a reduced separation efficiency similar to values of the initially unloaded filter. With an increasing amount of glass spheres, the separation of larger particles due to inertia and interception improves, causing the MPPS to shift towards a smaller particle size.

Further research on this topic is needed concerning the influence of the filter medium’s structural properties on the observed results. The wettability of the filter medium, as well as the transferability to filter media with fundamentally different core structures, e.g., depth filters, would be of specific interest. 

## Figures and Tables

**Table 1:** Analysis of drainage in total amounts.

Filter	Salt in Drainage/mg	Glass in Drainage/mg
unloaded	0	0
salt-laden	19.2	0
Salt–glass-laden 1:2	23.4	0
glass-laden	0	0

## Data Availability

Data made available upon request via corresponding e-mail.
